# A Global Analysis of Kinase Function in *Candida albicans* Hyphal Morphogenesis Reveals a Role for the Endocytosis Regulator Akl1

**DOI:** 10.3389/fcimb.2018.00017

**Published:** 2018-02-08

**Authors:** Hagit Bar-Yosef, Tsvia Gildor, Bernardo Ramírez-Zavala, Christian Schmauch, Ziva Weissman, Mariel Pinsky, Rawi Naddaf, Joachim Morschhäuser, Robert A. Arkowitz, Daniel Kornitzer

**Affiliations:** ^1^B. Rappaport Faculty of Medicine, Technion – Israel Institute of Technology, Rappaport Institute for Research in the Medical Sciences, Haifa, Israel; ^2^Institut für Molekulare Infektionsbiologie, Universität Würzburg, Würzburg, Germany; ^3^Centre National de la Recherche Scientifique, Institut National de la Santé et de la Recherche Médicale, Institute Biology Valrose, Université Côte d'Azur, Nice, France

**Keywords:** *Candida albicans*, hyphae, endocytosis, Pan1, functional genomics

## Abstract

The human pathogenic fungus *Candida albicans* can switch between yeast and hyphal morphologies as a function of environmental conditions and cellular physiology. The yeast-to-hyphae morphogenetic switch is activated by well-established, kinase-based signal transduction pathways that are induced by extracellular stimuli. In order to identify possible inhibitory pathways of the yeast-to-hyphae transition, we interrogated a collection of *C. albicans* protein kinases and phosphatases ectopically expressed under the regulation of the TETon promoter. Proportionately more phosphatases than kinases were identified that inhibited hyphal morphogenesis, consistent with the known role of protein phosphorylation in hyphal induction. Among the kinases, we identified *AKL1* as a gene that significantly suppressed hyphal morphogenesis in serum. Akl1 specifically affected hyphal elongation rather than initiation: overexpression of *AKL1* repressed hyphal growth, and deletion of *AKL1* resulted in acceleration of the rate of hyphal elongation. Akl1 suppressed fluid-phase endocytosis, probably via Pan1, a putative clathrin-mediated endocytosis scaffolding protein. In the absence of Akl1, the Pan1 patches were delocalized from the sub-apical region, and fluid-phase endocytosis was intensified. These results underscore the requirement of an active endocytic pathway for hyphal morphogenesis. Furthermore, these results suggest that under standard conditions, endocytosis is rate-limiting for hyphal elongation.

## Introduction

Phosphorylation, the most prevalent post-translational modification of cellular proteins, can modify protein conformation or protein-protein interactions, and as a consequence can alter the enzymatic activity of proteins, their localization, or their stability. Protein phosphorylation is essential to signal transduction, e.g., of extracellular stimuli to cellular proliferation or differentiation programs. The central role that kinases and phosphatases play in cellular regulation is reflected in their prevalence: kinases constitute about 2 percent of genes in both the human and the budding yeast (*S. cerevisiae*) genomes (Hunter and Plowman, 1997; Manning et al., 2002).

One of the many developmental processes where protein phosphorylation was shown to play a key role is the yeast to hyphae transition in the pathogenic yeast *Candida albicans. C. albicans* is the most common systemic fungal pathogen in developed countries, responsible for several hundred thousand deaths each year, chiefly among immunocompromised patients (Brown et al., 2012). Like several other fungal pathogens, *C. albicans* is able to assume different morphologies under different conditions (Sudbery et al., 2004). Whereas in normal growth medium it proliferates as yeast cells, increased temperature and exposure to stimuli such as serum or N-acetylglucosamine induces *C. albicans* to form hyphae. The signal transduction pathways that mediate hyphal induction have been thoroughly explored, and found to include transmission of extracellular signals via Ras to both protein kinase A and the MAP kinase cascade (Leberer et al., 1996; Feng et al., 1999; Biswas and Morschhäuser, 2005; Maidan et al., 2005). These signaling pathways result in the activation of a number of transcription factors such as Efg1 (Stoldt et al., 1997) and Ume6 (Banerjee et al., 2008; Zeidler et al., 2009) and inactivation of the transcriptional repressor Nrg1, which needs to be removed from hyphal-specific gene (HSG) promoters in order to allow induction of hyphal morphogenesis (Braun et al., 2001; Murad et al., 2001; Lu et al., 2011). Beyond signaling pathways, effective hyphal development requires alterations in many cellular functions; for example, growth of hyphae requires an active endocytic pathway, possibly in order to counterbalance the loss of membrane and proteins by exocytosis at the hyphal tip (Shaw et al., 2011).

Alongside pathways for hyphal induction, there are also mechanisms that suppress hyphal morphogenesis, such as protein degradation via the ubiquitin ligases Ubr1 and SCF^CaCDC4^ of the transcription factors Cup9 and Ume6, respectively (Atir-Lande et al., 2005; Lu et al., 2014; Mendelsohn et al., 2017). With regard to protein phosphorylation, while its activating roles in hyphal morphogenesis have been extensively investigated, less attention has been given to possible negative functions of protein phosphorylation in this process. In order to globally interrogate the potential role of protein phosphorylation in the suppression of hyphal morphogenesis, we took advantage of a collection of all identifiable kinase and phosphatase genes, as well as kinase and phosphatase regulators, cloned under the inducible TETon promoter. Using this collection, we identified kinase Akl1 among several potential novel regulators of hyphal morphogenesis. *Candida albicans* Akl1 was found, like its *S. cerevisiae* homolog, to repress endocytosis via its effect on the epsin homolog Pan1, a scaffolding protein linking the actin cytoskeleton to sites of clathrin-mediated endocytosis (Wendland and Emr, 1998; Takahashi et al., 2006; Bradford et al., 2015; Sun et al., 2015). The results shown here further underscore the relation between hyphal morphogenesis and the endocytic pathway, and suggest that hyphal elongation can be regulated via modulation of endocytosis.

## Materials and methods

### Media

All experiments were performed in YEP medium (1% Difco Yeast Extract, 2% Difco Bacto-peptone) supplemented with L-Tryptophan (0.15 g/l) and uridine (0.05 g/l), and either 2% glucose (= YPD) or 2% maltose or 2% raffinose, as indicated. Fetal bovine serum (Biological Industries, Israel) was added to 10% where indicated. For induction of the TETon promoter, doxycycline was added to 50 μg/ml. Selection for transformation with plasmids carrying auxotrophic markers was carried out on YNB plates supplemented with each amino acid and uridine, adenine at 0.1 g/l except leucine (0.3 g/l), but lacking the precursor used for selection.

### Plasmid and strain construction

#### Plasmids

The kinase and phosphatase library under the regulation of the TETon promoter is based on a library described previously (Ramírez-Zavala et al., 2013), to which we added 9 new kinase clones. In addition, the previously published library did not contain phosphatases; here we added 49 phosphatase genes, for a total of 222 genes (Supplementary Table 1). The MKK2 S317A T321A allele is a predicted dominant-negative allele, based on the phenotype of the corresponding mutations in human MEK5 (S311A T315A), which abolish the activating phosphorylation sites of this MEKK (Sun et al., 2001). The PBS2 K233A allele was modeled after the equivalent allele of its closest human homolog, MEK1 K97A, which displays a dominant-negative phenotype (Seger et al., 1994). CLN3ΔC has a nonsense mutation at codon 332, removing the C-terminal 133 residues of *C. albicans* Cln3, and yielding an expected stabilized and hyperactive protein, similar to the *S. cerevisiae* CLN3 alleles DAF1-1 and WHI1-1 (Cross, 1988; Nash et al., 1988). KB2213 contains the open reading frame (ORF) of *AKL1* (−1 to + 2418) cloned HindIII-ApaI into KB1817 (Ofir et al., 2012). KB2301 contains *AKL1* (from −470 to +2418) cloned NotI-HindIII into pBES116 (Feng et al., 1999). KB2381 contains the *AKL1* ORF (−1 to + 2418) cloned EcoRV-KpnI under the *MAL2* promoter of pBES119 (Feng et al., 1999). KB2391 contains the *PAN1* ORF (−1 to +4188) cloned SmaI-XhoI into KB2083 (Ofir et al., 2012) cut EcoRV-SalI. KB2434 contains the 3′ end of the *PAN1* ORF (+268 to +4188) cloned BamHI-XhoI into KB2430, itself containing the GFPgamma + *URA3* fragment of pFA-GFPγ-URA3 (Zhang and Konopka, 2010) cloned XhoI-KpnI into pBSII-SK+ (Stratagene).

#### Strains

The *C. albicans* strains are listed in Table 1. KC553, KC554 were generated by PCR-mediated deletion of *AKL1* alleles (Noble and Johnson, 2005). KC651 contains the *MAL2* promoter of plasmid pFA-URA3-MAL2p (Gola et al., 2003) integrated upstream of the *UME6* open reading frame by PCR-targeted recombination. KC816, KC817 contain the vector plasmid KB1817 and KB2213 (*HIS1 MAL2-AKL1*) respectively. KC824, KC825 contain the vector plasmid BES116 and KB2301 (*AKL1*) respectively. KC840, KC841 contain the vector plasmid BES116 and KB2381 (*URA3 MAL2-AKL1*). KC850, KC851 contain the vector plasmid pBES119 and KB2381, respectively. KC857 and KC869 contain plasmid KB2391. KC862 contains plasmids KB2213 and pBES119, while KB863 contains KB2213 and KB2391. KC868 contains vector plasmid BES116. KC908 and KC909 were constructed by integrating GFP of plasmid KB2434 downstream of *PAN1*. KC919, KC920 contain vector plasmid KB1817 and plasmid KB2213, respectively. KC983 and KC984 were constructed by integrating *PAN1-3xHA* into a *PAN1* allele using KB2391 digested with HindIII, which cuts in the middle of the *PAN1* ORF. KC987, KC988 contain vector plasmid BES119 and KB2391, respectively. KC1062, KC1063 contain vector plasmid KB1817 and plasmid KB2213, respectively.

**Table 1:** List of *C. albicans* strains.

**Name**	**Genotype**	**Origin**
KC271=SN78	*ura3Δ/ura3Δ leu2Δ/leu2Δ*	Noble and Johnson, 2005
KC274=SN148	*ura3Δ/ura3Δ his1Δ/his1Δ leu2Δ/leu2Δ arg4Δ/arg4Δ*	Noble and Johnson, 2005
KC445	*ura3Δ/ura3Δ ume6Δ*::hisG/*ume6Δ*::hisG	Mendelsohn et al., 2017
KC553	KC274 *AKL1/akl1Δ::LEU2*	This work
KC554	KC274 *akl1Δ::HIS1/akl1Δ::LEU2*	This work
KC651	KC271 *UME6/MAL2p::UME6 URA3*	Mendelsohn et al., 2017
KC816	KC274 *ADE2/ade2::*<*HIS1>*	This work
KC817	KC274 *ADE2/ade2::*<*HIS1 MAL2p-AKL1>*	This work
KC824	KC554 *ADE2/ade2::*<*URA3>*	This work
KC825	KC554 *ADE2/ade2::*<*URA3 AKL1>*	This work
KC840	KC274 *ADE2/ade2::*<*URA3>*	This work
KC841	KC274 *ADE2/ade2::*<*URA3 MAL2p-AKL1>*	This work
KC850	KC445 *ADE2/ade2::*<*URA3>*	This work
KC851	KC445 *ADE2/ade2::*<*URA3 MAL2p-AKL1>*	This work
KC857	KC554 *ADE2/ade2::*<*URA3 MAL2p-PAN1-3xHA>*	This work
KC862	KC274 *ade2::*<*HIS1 MAL2p-AKL1>/*<*ade2::URA3>*	This work
KC863	KC274 *ade2::*<*HIS1 MAL2p-AKL1>/*<*ade2::URA3 MAL2p-PAN1>*	This work
KC868	KC274 *ADE2/ade2::*<*URA3>*	This work
KC869	KC274 *ADE2/ade2::*<*URA3 MAL2p-PAN1-3xHA>*	This work
KC908	KC274 *PAN1/PAN1-GFP::URA3*	This work
KC909	KC554 *PAN1/PAN1-GFP::URA3*	This work
KC919	KC651 *ADE2/ade2::*<*HIS1>*	This work
KC920	KC651 *ADE2/ade2::*<*HIS1 MAL2p-AKL1>*	This work
KC983	KC274 *PAN1/PAN1-3xHA::URA3*	This work
KC984	KC554 *PAN1/PAN1-3xHA::URA3*	This work
KC987	KC445 *ADE2/ade2::*<*URA3>*	This work
KC988	KC445 *ADE2/ade2::*<*URA3 MAL2p-PAN1-3xHA>*	This work
KC1062	KC983 *ADE2/ade2::*<*HIS1>*	This work
KC1063	KC983 *ADE2/ade2::*<*HIS1 MAL2p-AKL1>*	This work

### Library screening

WO-1 cells transformed with the TETon kinase library (Ramírez-Zavala et al., 2013) were grown overnight at 30°C in YPD in 96-well plates, then diluted 1:5 in YPD + 10% fetal bovine serum, 50 μg/ml doxycycline, in 96-well plates (150 μl/well) and incubated at 30°C for 5.5 h. Cells were then fixed with 4% formaldehyde and observed microscopically for morphology. Morphology was scored between −4 (no hyphae at all) and +4. The experiment was repeated 3 times; Supplementary Table 1 indicates the average score for each clone.

### Microscopy

DIC micrographs were taken with a Zeiss AxioImager.M1 equipped with 40X and 100X objectives. For measurements of hyphal length, cells were fixed with 70% ethanol, then pelleted and resuspended in 2% H_2_SO_4_ to reduce cell clumping. Lengths were measured with the Axiovision software measuring function. The person performing the measurements was blinded to the identity of the cultures. Statistical analysis of cell size distributions (*t*-test, regression analysis) was performed using Microsoft Excel's analysis package.

For visualization of Pan1-GFP, cells were fixed by addition of formaldehyde to 2% and incubating 10 min at 30°C in a roller. The cells were then washed twice with phosphate-buffered saline (PBS), separated by repeated aspiration through a 29G needle and resuspended in Vectashield mounting medium. A Zeiss LSM700 confocal system was used to gather images. The micrographs in **Figure 6** represent a maximum intensity projection of a full fluorescence Z-stack overlaid upon a DIC micrograph. Quantitation of the Pan1-GFP signals as a function of the distance from the hyphal tip was achieved using the Bitplane Imaris 9.0.1 software's spots detection function. The threshold was set such that non-tagged control cells displayed 1 or 2 spots at most, whereas tagged cells usually displayed between 30 and 120 spots.

### Fluid-phase endocytosis assay

Lucifer Yellow (LY) uptake was used as a measure of fluid-phase endocytosis (Basrai et al., 1990; Dulic et al., 1991). Overnight cultures were diluted to the appropriate medium and grown 2–3 h to an OD_600_ of 0.1. For each assay, 10 ml of the culture was spun down (3,500 g, 5′) and resuspended in 0.9 ml of fresh medium. 0.1 ml of Lucifer Yellow CH (LY; Sigma), 40 mg/ml in water, was added and the cultures were incubated another 1 h while shaking. The cells were then spun down, resuspended in 1 ml ice-cold stop buffer (50 mM Succinate-NaOH, pH 7.5, 100 mM NaCl, 10 mM NaN_3_, 10 mM MgCl_2_), and spun down again a total of 6 times. The final pellet was resuspended in 1 ml lysis buffer (50 mM Tris-Cl Ph 7.5 m 10 mM 2-mercaptoethanol, 2,000 U lyticase (Sigma)/ml) and incubated 15′ at 37°C. LY fluorescence was quantitated using a Tecan Infinite M200 PRO spectrofluorimeter with 426 nm excitation and 550 nm emission. Fluorescence was calibrated with free LY in the same buffer. To normalize LY uptake to cell amount, the protein amount was measured by the Bradford method (Bio-Rad protein assay dye reagent), using 0.2 ml of the same extract used for LY quantitation. Bovine serum albumin (Sigma) was used for calibration of the protein assay. Since even the normalized LY uptake was highly sensitive to incubation conditions and LY batch, only assays carried out together could be directly compared.

### Pan1 protein analysis

For Western blotting, 1.5 ml culture samples (~1.5 O.D_600_ cells) were spun down, resuspended in 0.5 ml 0.25M NaOH, 1% 2-mercaptoethanol, incubated on ice 10 min, then precipitated on ice with 7% trichloroacetic acid. The pellets were washed with ice-cold acetone and resuspended in gel loading buffer, separated by SDS-PAGE (BioRad 4–20% prepoured gels), transferred to nitrocellulose, and detected with HA.11 (Covance) and anti-β-actin (Sigma). ^35^S-methionine pulse-chase analysis was carried out essentially as described (Kornitzer, 2002). Briefly, 10 ml cultures (O.D_600_ ~ 1) were washed twice with 0.4 ml YNB medium + 2% maltose and resuspended in the same medium + 1 mCi Express labeling mix (Perkin-Elmer), incubated 7 min at 30°C, then spun down and resuspended in 2.5 ml synthetic complete medium + 10 mM methionine, cysteine. 0.6 ml aliquots retrieved at indicated times were treated with 0.25M NaOH, 1% 2-mercaptoethanol, then precipitated on ice with 7% trichloroacetic acid. The pellets were washed with acetone, resuspended in 2.5% SDS, 5 mM EDTA, and equal amounts of incorporated radioactivity were immunoprecipitated by diluting the extract at least 10-fold in i.p. buffer (50 mM Na-Hepes pH 7.5, 0.15 M NaCl, 5 mM Na-EDTA, 1% Triton X-100, 1 mM PMSF, HA.11 (Covance) 1 μg/m) and adding Protein A sepharose CL-4B (GE Healthcare).

### RNA analysis

The “hot phenol” method (Collart and Oliviero, 2001) was used to extract RNA. Growth and mRNA analysis was performed as described in Ofir et al. (2012).

## Results

### Identification of genes that suppress hyphal morphogenesis

A collection of over 200 protein kinases, phosphatases, and some ancillary factors (see Methods), expressed in the *C. albicans* WO-1 strain under the TETon promoter, was screened for genes that inhibited hyphal morphogenesis. For each gene, two independently transformed strains were assayed by diluting an overnight culture into YPD + 10% fetal calf serum at 30°C, and monitoring hyphal morphogenesis (Supplementary Table 1). Table 2 lists 32 genes that consistently reduced hyphal formation to greater or lesser extent, 16 kinases or kinase-associated proteins (e.g., cyclins) and 16 phosphatases or phosphatase-associated proteins. Since the collection contained only 22% phosphatases (and associated proteins), the phosphatase representation in the hyphal morphogenesis-suppressing genes was significantly higher than expected (binomial probability = 3 × 10^−4^), consistent with the well-studied role of kinases in the signal transduction pathways leading to hyphal morphogenesis. Specific phosphatases in this set that were previously shown to affect hyphal morphogenesis include PSR1 (Elson et al., 2009), PTC1 (Hanaoka et al., 2008), SHP1 and GLC7 (Hu et al., 2012), PPG1 (Albataineh et al., 2014), and PPH3 (Sun et al., 2011).

**Table 2:** Genes that suppress filamentation in YPD + 10% serum.

**Kinases (or kinase-associated proteins)**	**Phosphatases (or phosphatase-associated proteins)**
***AKL1***	*MIH1*
*BUD32*	***PTC5***
***KIS1*** (Snf1 complex scaffold)	***TPD3*** (PP2A scaffold)
***PCL5*** (Pho85 cyclin)	***PSR1***
***CLN3***Δ**C** (Cdc28 cyclin)	***PTC1***
***PBS2***^K233A^	*PTC4*
*HSL1*	**19.3302** (PP-I targeting subunit)
***IKS1***	*RTS1* (PP2A regulator)
***KCS1***	***RRD2*** (PP2A regulator)
*RIM15*	*GLC7* (phosphatase)
***SNF4*** (Snf1 complex activator)	***PPG1***
***SOL1*** (Cdc28 inhibitor)	*PTP3*
***MKK2***^S317A T321A^	*SHP1* (possible PP-I regulator)
*GCN2*	*PPH3*
***VPS15***	*PPQ1*
***CCL1*** (Kin28 cyclin)	***PPZ1***

*Boldface, genes with both clones scoring at least −1 in hyphal formation*.

Regular face, genes with one clone scoring at least −1 and the other at least −0.5

Among the kinases and associated proteins, a number of genes previously implicated in hyphal morphogenesis were also found, e.g., Snf4 (Uhl et al., 2003) which, together with the upstream activating kinase Sak1 and the β-subunits Kis1 and Kis2, regulates Snf1 kinase activity (Corvey et al., 2005; Ramirez-Zavala et al., 2017); the Pho85 cyclin Pcl5, a regulator of the transcription factor Gcn4 (Gildor et al., 2005); the Cdc28/Cdk1 cyclin Cln3 (Bachewich and Whiteway, 2005; Chapa Y Lazo et al., 2005; Zeng et al., 2012; Mendelsohn et al., 2017) and the Cdc28/Cdk1 inhibitor Sol1 (Atir-Lande et al., 2005); the MAPKK Pbs2 (Arana et al., 2005); and the Nim1 kinase Hsl1 (Wightman et al., 2004; Umeyama et al., 2005). Other genes have homologs involved in hyphal morphogenesis in filamentous fungi, e.g., Bud32, a homolog of *Aspergillus nidulans* pipA (Kempf et al., 2013); and Rim15, a homolog of *N. crassa* stk-12 (Watters et al., 2011).

### The kinase Akl1 suppresses hyphal morphogenesis

**Figure 1:**
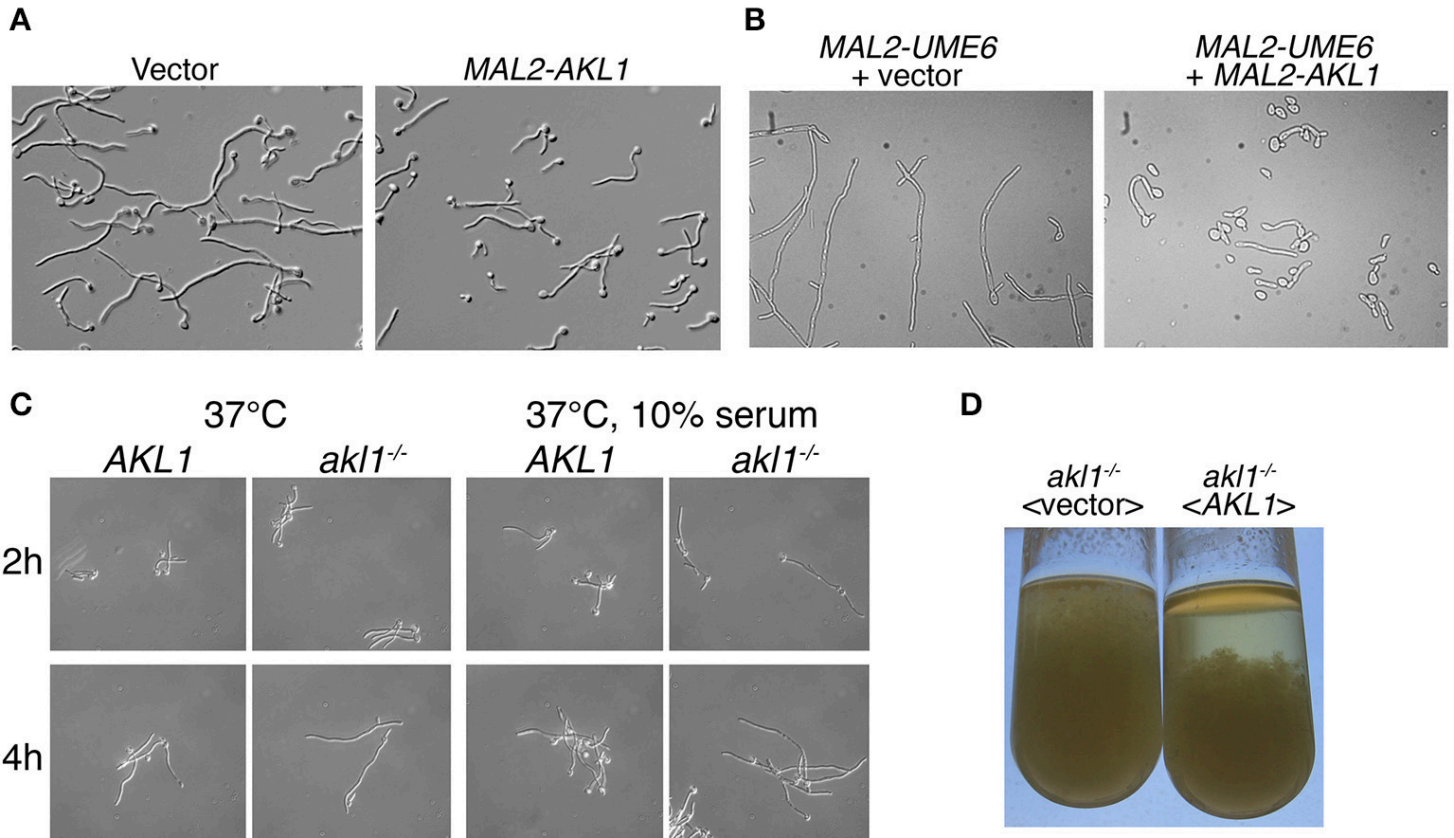
Hyphal morphogenesis in cells overexpressing *AKL1* or deleted for *AKL1*. **(A)** Micrographs of wild-type cells carrying a vector plasmid (KC840) vs. the *MAL2-AKL1* construct (KC841). Overnight cultures were diluted in YEP + 2% maltose + 10% FCS and incubated 3 h at 37°C. **(B)** A strain containing a *MAL2* promoter upstream of the *UME6* ORF was transformed with either a vector plasmid (KC919) or the *MAL2-AKL1* construct (KC920). An overnight culture was diluted in YEP + 2% maltose and incubated for 7 h at 30°C. **(C)** Comparison of hyphal induction in wild-type (KC274) and *akl1*^−/−^ deletion (KC554) under the indicated conditions. Overnight cultures were diluted in YPD with or without 10% FCS. **(D)** The indicated *akl1*^−/−^ deletion and reintegrant strains (KC824 and KC825) were grown overnight at 37°C in YPD + 10% serum. The tubes were vigorously vortexed, then left to sediment for 30 min.

One of the kinases that gave the strongest suppression of hyphae formation, Akl1, had not previously been identified as being involved in hyphal morphogenesis. We thus first confirmed the effect of Akl1 in the standard laboratory strain SC5314. Since our TETon promoter is less active in SC5314 than in WO-1 background, we switched to an alternative overexpression system, by placing the *AKL1*/19.5357 open reading frame under the regulation of the maltose-induced *MAL2* promoter, which yielded a 4–5-fold increase in *AKL1* mRNA (see below, **Figure 8**), and expressing it in the CAI4 strain background (Fonzi and Irwin, 1993). *AKL1* suppressed hyphal growth in this system as well (Figure 1A).

Hyphal morphogenesis depends on activation of a number of signal transduction pathways that culminate with the induction of the *UME6* gene (Banerjee et al., 2008; Zeidler et al., 2009). Ume6 is a transcription factor that activates genes essential for hyphal morphogenesis, and it is itself both necessary and sufficient for hyphal morphogenesis (Carlisle et al., 2009). In order to distinguish whether Akl1 interferes with hyphal induction by blocking *UME6* induction, or by interfering with cellular functions induced by Ume6, we artificially induced hyphal morphogenesis by ectopically expressing Ume6 under the *MAL2* promoter, with or without co-expression of *AKL1* under *MAL2* (Figure 1B). Co-overexpression of *AKL1* significantly reduced the extent of hyphal elongation induced by Ume6 overexpression, suggesting that Akl1 exerts its effect either post-transcriptionally on Ume6 itself, or by interfering with cellular mechanisms required for hyphal morphogenesis.

Since overexpression of *AKL1* suppressed hyphal growth, we next tested the phenotype of the deletion of *AKL1* on morphogenesis. We deleted both alleles of *AKL1*, followed by reintegration of a wild-type allele in the deletion strain. Compared to the wild-type strain, the deletion strain exhibited more robust hyphal growth, best detected under sub-optimal hyphal induction conditions, such as a shift to 37°C in regular YPD medium, but also visible in optimal hyphal induction conditions, 37°C + 10% serum (Figure 1C). This difference in hyphal induction was also visible after overnight growth of the different strains under hyphal induction conditions: when comparing the *akl1*^−/−^ deletion strain with the same strain containing a reintegrated wild-type *AKL1* allele, longer hyphae were visible in the *akl1*^−/−^ deletion strain. Macroscopically, this was expressed as a non-sedimenting mycelium that filled the whole tube. In contrast, the reintegrant strain sedimented rapidly, as usually observed with hyphal *C. albicans* cultures (Figure 1D).

### Characterization of the effect of Akl1 on hyphal morphogenesis

**Figure 2:**
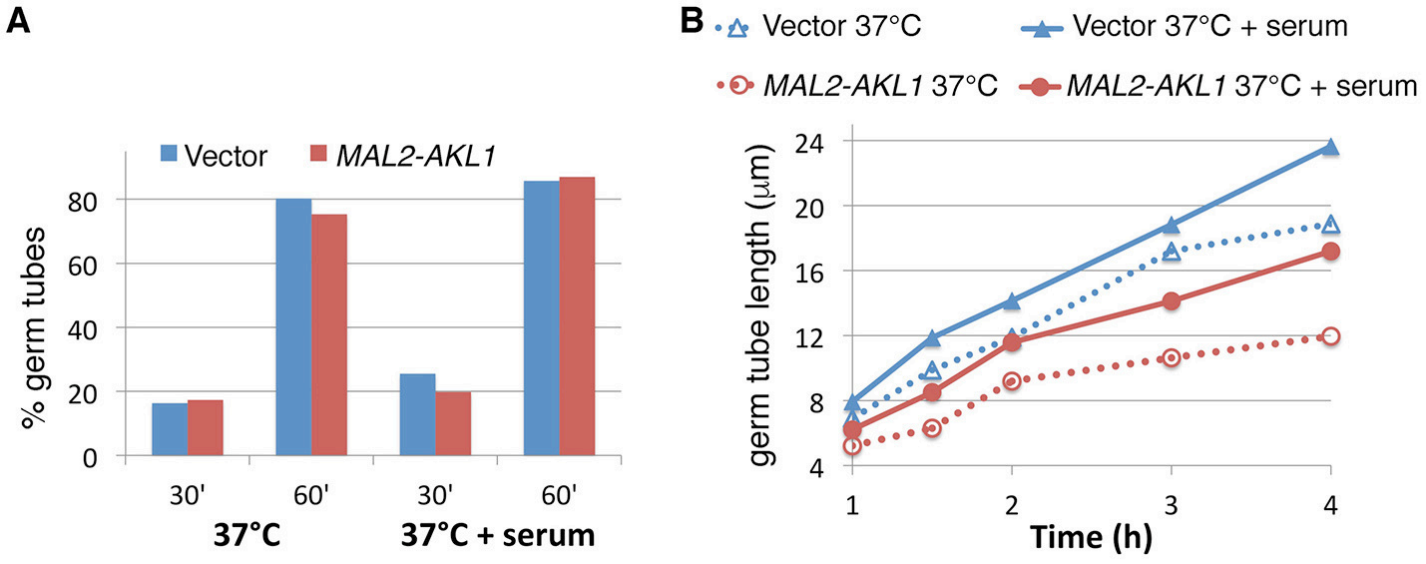
*AKL1* overexpression suppresses the rate of hyphal elongation. **(A)** Wild-type cells containing a *URA3* vector plasmid (KC840) or a *URA3 MAL2-AKL1* plasmid (KC841) were grown overnight in YEP + 2% raffinose, then diluted in YEP + 2% maltose at 37°C, with or without 10% serum, as indicated. Samples were retrieved and fixed at the indicated times and the proportion of germ tubes was determined by microscopic observation. Six hundred cells were counted in each culture. **(B)** Length of germ tubes was measured in the same cultures as for **(A)**. Hundred cells were measured for each condition. The differences between each vector and *MAL2-AKL1* pair were all highly significant by Student's *t*-test (*p* < 10^−4^).

**Figure 3:**
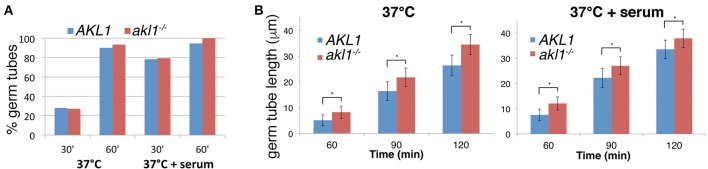
Hyphal elongation rate is increased in the *akl1*^−/−^ mutant. **(A)** Wild-type (KC 274) vs. *akl1*^−/−^ (KC554), germ tube induction. An overnight culture was diluted in YPD at 37°C, with or without 10% serum, as indicated. Samples were fixed at the indicated times and the proportion of germ tubes was determined by microscopic observation. Six hundred cells were counted in each culture. **(B)** Length of germ tubes was measured in the same cultures as for **(A)**. Hundred cells were measured for each condition. The error bars indicate the standard deviations. All differences were highly significant by Student's two-tailed *t*-test (^*^p < 10^−15^).

Microscopic observation of cells after a few hours' growth in hypha-inducing conditions showed shorter hyphae in cells overexpressing *AKL1* (Figure 1A). Conversely, the *akl1*^−/−^ mutant displayed longer hyphae, compared to the wild-type (Figure 1C). In order to quantitate these effects, and to dissociate potential effects of Akl1 on hyphal germ tube induction from effects on hyphal elongation, wild-type cells overexpressing *AKL1* under the *MAL2* promoter were subjected to hyphal induction by shift to 37°C, without or with addition of 10% serum, and germ tube proportion and length were measured at fixed time intervals after induction (Figure 2). In a parallel experiment, cultures of the wild-type and mutant *AKL1* strains were similarly compared (Figure 3), as well as of the *AKL1* mutant vs. reintegrant strain (Supplementary Figure 1). Analysis of the data indicates that the extent of germ tube induction was unaffected by Akl1 activity (Figures 2A, 3A). In contrast, the rate of germ tube elongation was significantly affected by Akl1 activity. In cells overexpressing *AKL1*, hyphal extension rates between 1 and 4 h after shift to 37°C were reduced by 29 and 44% in medium with or without serum, respectively (Figure 2B). In the absence of *AKL1*, conversely, an increase in hyphal extension rate was detected. However, the effect was biphasic: within the first hour after shift to 37°C, in both the presence and absence of serum, an increase of 60% in hyphal length was detected in the *akl1*^−/−^ strain (Figure 3B, 60 min time point). During the second hour after induction, in the presence of serum, no additional increase in the difference between *akl1*^−/−^ and *AKL1* hyphal lengths was detected, indicating that the hyphal extension rates had equalized, whereas in the absence of serum, the *akl1*^−/−^ strain still exhibited a 24% faster hyphal elongation rate (Figure 3, 60–120 min time points). Notably, deletion of *AKL1*, or overexpression of *AKL1* under the *MAL2* promoter, had no detectable impact on growth at 37°C (Supplementary Figure 2).

### Effect of Akl1 on endocytosis

**Figure 4:**
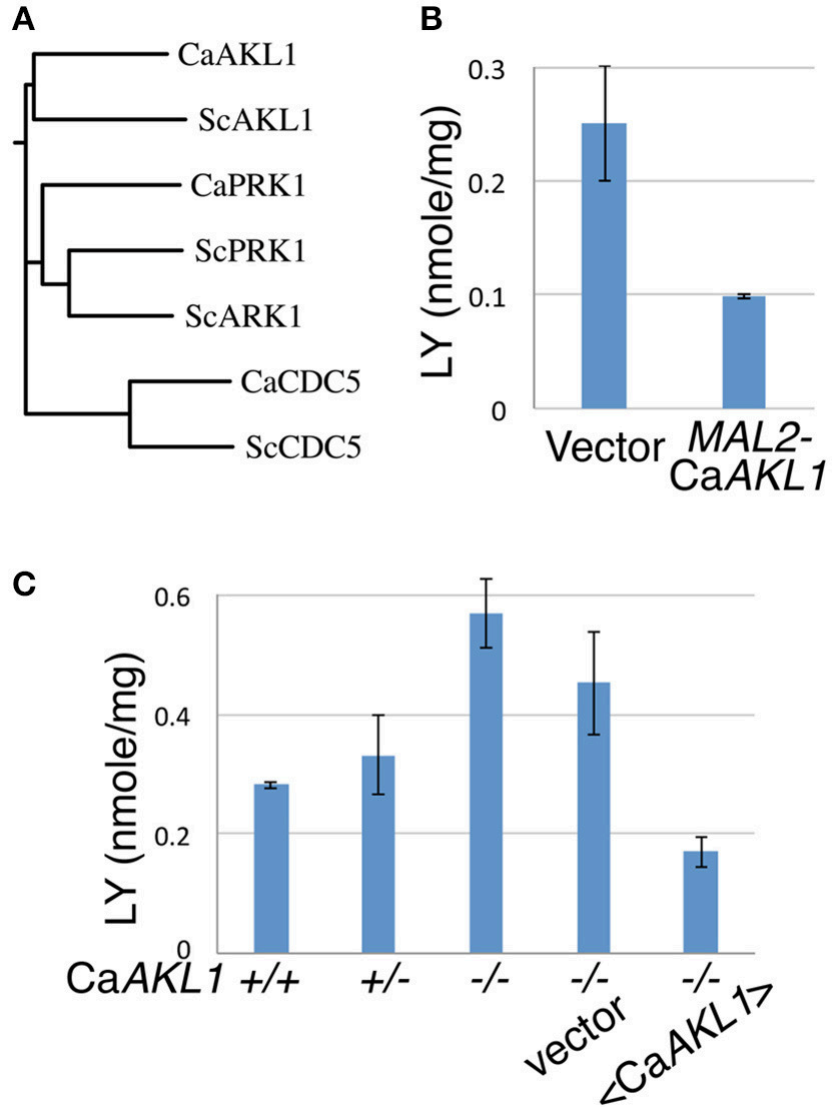
CaAkl1 is a repressor of fluid-phase endocytosis. **(A)** Homology tree of the Polo and Ark/Prk kinases of *S. cerevisiae* and *C. albicans* aligned using T-COFFEE (Di Tommaso et al., 2011). **(B)** LY uptake in wild-type cells overexpressing *AKL1* under the *MAL2* promoter (KC 817) vs. vector plasmid (KC816). **(C)** LY uptake in wild-type (KC274) cells, the *AKL1*^+/−^ heterozygote (KC553), the *akl1*^−/−^ deletion homozygote (KC554), *akl1*^−/−^ with vector plasmid (KC824) or with *AKL1* plasmid (KC825). Overnight cultures were shifted from YEP + 2% raffinose to YEP + 2% maltose **(B)** or diluted in YPD **(C)** and incubated at 37° for 2.5 h before carrying out the assays. The error bars indicate the standard deviation between three independent cultures.

Based on sequence homology, *C. albicans* Akl1 is related to the Ark/Prk family of kinases (Smythe and Ayscough, 2003), and is more distantly related to the Polo-like kinases (Cdc5). The *S. cerevisiae* genome contains two Ark/Prk homologs and a single Akl1 homolog, whereas the *C. albicans* genome contains one homolog of each (Figure 4A). Akl1 was shown to reduce fluid-phase endocytosis when overexpressed from a high-copy plasmid in *S. cerevisiae* (Takahashi et al., 2006). We therefore tested whether overexpression of Akl1 would likewise reduce fluid-phase endocytosis in *C. albicans*. LY uptake was monitored in cells overexpressing Akl1 under the *MAL2* promoter vs. control cells, and in wild-type vs. *akl1*^−/−^ cells. LY uptake was reduced in cells overexpressing Akl1 (Figure 4B), and it was enhanced in *akl1*^−/−^ cells, compared to the wild-type and reintegrant strains (Figure 4C; we noticed that reintegration of *AKL1* at the *ADE2* locus resulted, both here and in Supplementary Figure 1, in a phenotype consistent with partial overexpression, which could be due to a gene position effect). We thus conclude that Akl1 functions as a repressor of fluid-phase endocytosis.

However, we noted that the effect of Akl1 on LY uptake was reduced at 30°C compared to 37°C (data not shown). Since hyphal morphogenesis is induced in the control cells at 37°C, and suppressed by Akl1 activity, it was possible that hyphae are more active in endocytosis than yeast cells, and that Akl1 indirectly suppresses endocytosis by suppressing hyphal morphogenesis. To rule out this possibility, we overexpressed Akl1 in the *ume6*^−/−^ mutant, which is unable to form hyphae (Banerjee et al., 2008; Zeidler et al., 2009). In these cells as well, Akl1 overexpression caused a decrease in LY uptake (Supplementary Figure 3), indicating that Akl1's effect on endocytosis is not an indirect consequence of its effect on hyphal morphogenesis.

### Akl1 induces post-translational modification of *C. albicans* Pan1

**Figure 5:**
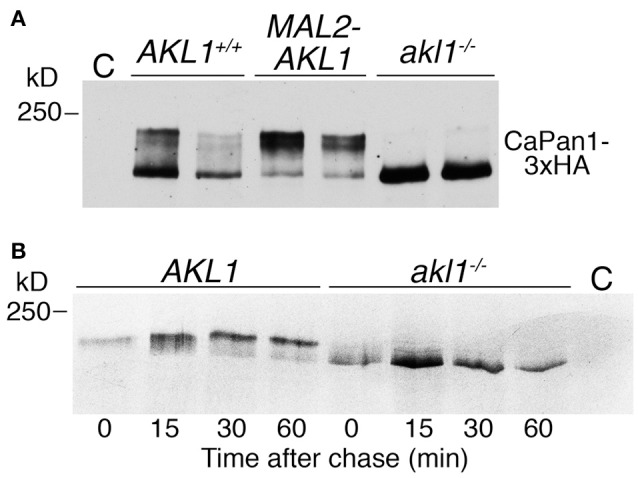
Akl1 induces modification of Pan1. **(A)** Western blot analysis of Pan1-3xHA expressed under its endogenous promoter in *AKL1*^+/+^ (KC1062) and *akl1*^−/−^ (KC984) cells, or in *AKL1*^+/+^ cells expressing *AKL1* under the *MAL2* promoter (KC1063). Cells (2 clones of each genotype) were grown overnight in YEP-raffinose, then shifted to YEP-2% maltose for 4 h. Extracts were migrated on a 6% SDS-PAGE gel. **(B)** Pulse-chase analysis of Pan1-3xHA expressed under the *MAL2* promoter in *AKL1*^+/+^ (KC 869) and *akl1*^−/−^ cells (KC857). Cells were grown overnight in YEP-raffinose, then shifted to YEP-2% maltose for 3 h before pulse-labeling with ^35^S-methionine, followed by a chase with cold methionine for the indicated amounts of time. C = no-tag control.

Pan1 is a yeast clathrin-mediated endocytosis scaffold protein, homologous to mammalian Intersectin. Its closest *C. albicans* homolog was previously suggested to be essential for endocytosis in this organism as well (Martin et al., 2007). Since there was evidence that *S. cerevisiae* Akl1 phosphorylates Pan1 *in vivo* (Takahashi et al., 2006), we tested whether *C. albicans* Pan1 was differentially post-translationally modified in the absence of *AKL1*, or under *AKL1* overexpression. Migration of epitope-tagged Pan1 on SDS-PAGE followed by Western blotting showed a main band with a slower-migrating “smear” above it, typical of phosphoryated species (Figure 5A). The slower-migrating species were not visible in the *akl1*^−/−^ strain, and conversely, they represented a larger fraction of total Pan1 in cells overexpressing *AKL1* (Figure 5A). Furthermore, the steady-state levels of Pan1 were elevated in the absence of *AKL1* (Supplementary Figure 4). In order to measure the stability of Pan1 as well as to follow the dynamics of the appearance of these slower-migrating species, the cells were subjected to pulse-and-chase analysis with ^35^S-methionine (Figure 5B). Whereas no change in the main Pan1 band was visible in the *akl1*^−/−^ strain, a slower-migrating species gradually appeared in the wild-type strain. This slower-migrating species reached a steady-state level after about 30 min, where it constituted a majority of Pan1. However, we detected no effect of the *AKL1* genotype on Pan1 stability.

Since these experiments were performed in maltose medium, we also tested whether the *AKL1* genotype affected the Pan1 protein in glucose medium. As shown in Supplementary Figure 5, a similar decrease in slower-migrating species of Pan1 was detected in the *akl1*^−/−^ mutant grown in glucose, but the effect on Pan1 steady-state levels was much reduced in this medium.

### Akl1 affects *C. albicans* Pan1 localization

**Figure 6:**
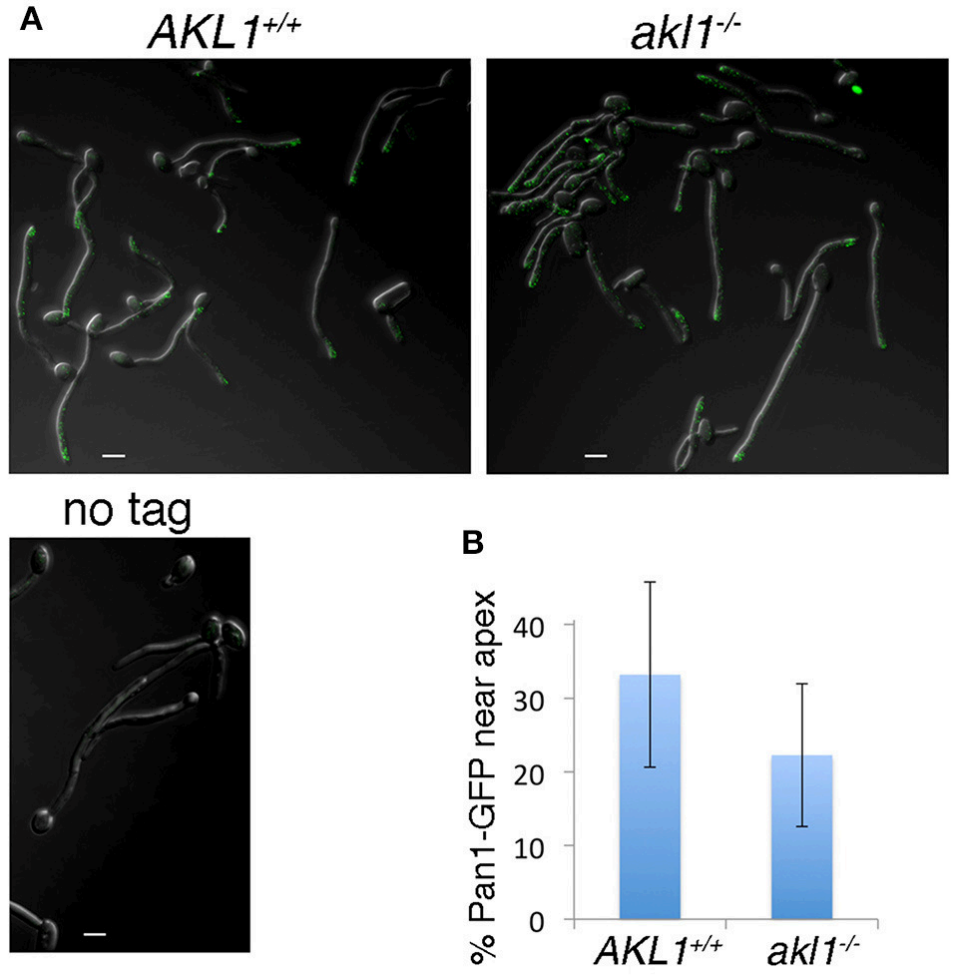
Pan1 patches are delocalized in the absence of Akl1. Pan1-GFP is expressed under its endogenous promoter in the wild-type (KC908) or *akl1*^−/−^ cells (KC909), or the non-tagged wild type strain. **(A)** The GFP signal was visualized by confocal microscopy in cells fixed 2 h after hyphal induction by a shift to 37°C in YPD + 10% FCS. **(B)** The graph represents the percentage of Pan1-GFP spots within 2 μm of the hyphal tip, relative to the total number of spots in that hypha. *N* = 27 (*AKL1*), 30 (*akl1*^−/−^), *p* < 10^−3^.

*S. cerevisiae* Pan1 was shown to localize to dynamic patches on the cell surface, together with other proteins associated with clathrin-mediated endocytosis (Wendland and Emr, 1998; Kaksonen et al., 2003, 2005). In *C. albicans* hyphal cells, the Pan1 homolog was found to be distributed along the length of the hyphae, but with higher concentration toward the apex (Martin et al., 2007). We assayed whether Akl1 influences *C. albicans* Pan1 localization by visualizing a Pan1-GFP fusion protein in wild-type vs. *akl1*^−/−^ cells. Consistent with previous observations (Martin et al., 2007), we also find that Pan1-GFP is mostly localized sub-apically in wild-type cells. In *akl1*^−/−^ cells however, it is delocalized and is more broadly distributed along the length of the hyphae (Figure 6). This broader distribution might explain the more active endocytosis detected in the *akl1*^−/−^ cells (Figure 4).

### *PAN1* overexpression antagonizes the effect of *Akl1* overexpression on hyphal morphogenesis and endocytosis

**Figure 7:**
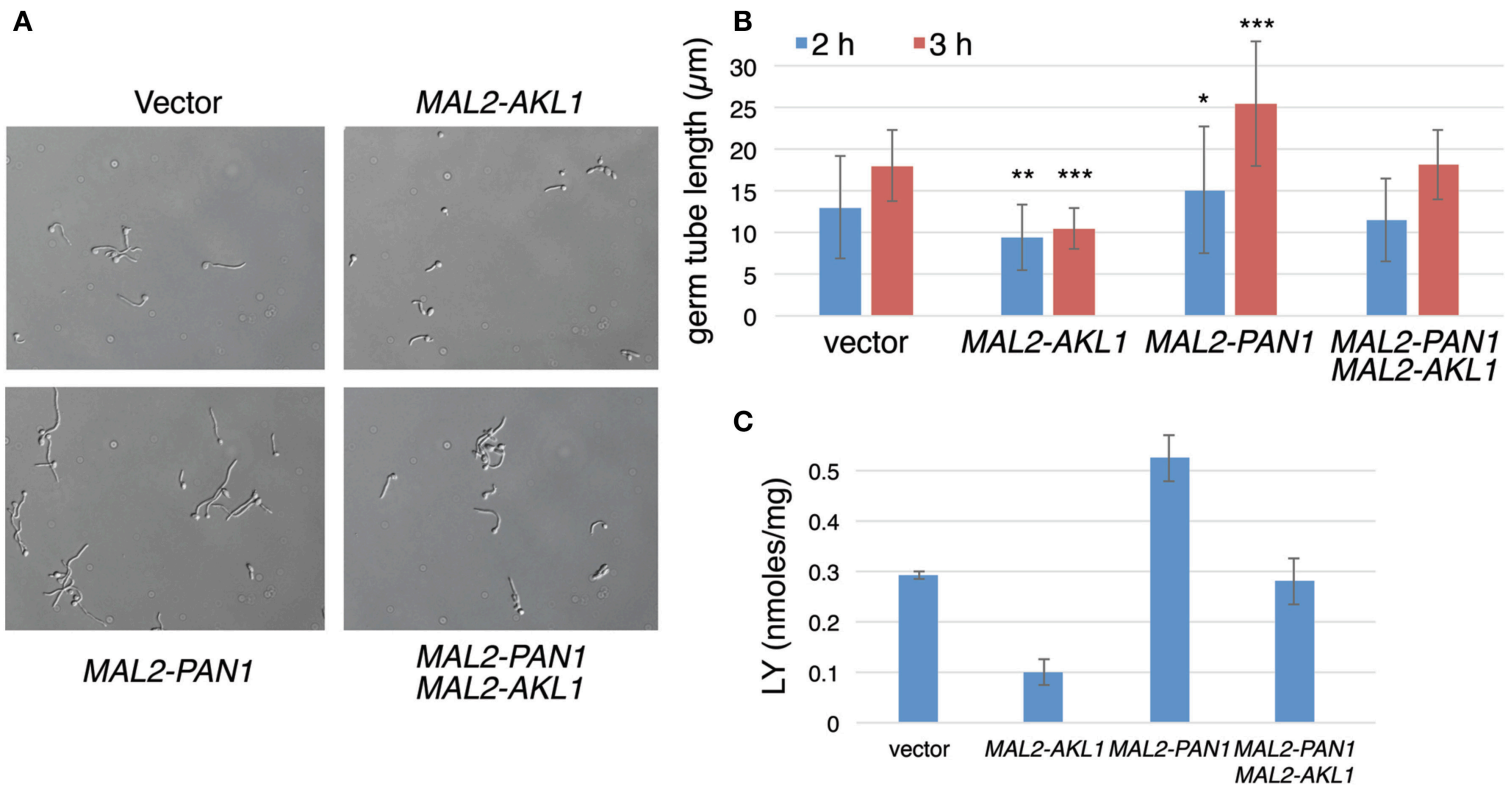
Pan1 is antagonistic to Akl1 for suppression of hyphal morphogenesis and endocytosis. Overnight cultures of wild-type cells containing the *MAL2-AKL1* construct (KC817) or the *MAL2-PAN1* construct (KC869) separately, or both plasmids together (KC863), or just the vector plasmid (KC868), were diluted in YEP-2% maltose at 37°C. **(A)** Micrographs of the four cultures after 3 h growth. **(B)** Measurement of 100 germ tubes each of the cultures after 2 and 3 h growth at 37°C. The bars indicate the average germ tube lengths, the error bars indicate the standard deviations, and the asterisks indicate statistical significance (Student's two-tailed *t*-test) of the difference for each individual culture compared to the vector control. ^*^p < 0.05, ^**^p < 10^−6^, ^***^p < 10^−15^. **(C)** LY uptake in the same transformants. The bars indicate the average of 3 cultures each, and the error bars indicate the standard deviations.

If reduction of Pan1 activity in endocytosis is responsible for the reduced hyphal morphogenesis in the presence of overexpressed Akl1, then it may be possible to suppress the effect of Akl1 on hyphal morphogenesis by increasing Pan1 activity. To test this, we co-overexpressed Pan1 and Akl1 alone and together, and monitored hyphal elongation upon shift to 37°C. As shown in Figure 7, the suppression of hyphal morphogenesis by Akl1 was canceled by co-overexpression of Pan1. This is consistent with the possibility that Pan1 is the main target of Akl1 in the hyphal morphogenesis suppression mechanism. The effects of the expression of these genes on the time of germ tube appearance was minor and could not account for the differences in hyphal elongation (Supplementary Figure 6).

Interestingly, overexpression of *PAN1* by itself led to faster hyphal elongation (Figures 7A,B). We therefore tested whether, similar to the *AKL1* deletion, *PAN1* overexpression also caused an increase in LY uptake. Cells that overexpress *PAN1* do in fact show higher levels of LY uptake (Figure 7C). *AKL1* overexpression caused reduction in LY uptake (Figure 7C), as shown before (Figure 4B), whereas expression of both genes together restored wild-type levels of LY uptake (Figure 7C). In order to dissociate the effect of Pan1 on endocytosis and on hyphal morphogenesis, we used the *ume6*^−/−^ mutant, which does not form hyphae, as before. We found that *PAN1* overexpression still increased LY uptake in the *ume6*^−/−^ mutant (Supplementary Figure 7), indicating that the effect of *PAN1* overexpression on endocytosis is not a consequence of its effect on hyphal elongation.

### *PAN1* and *AKL1* affect the expression of hyphal-specific genes

**Figure 8:**
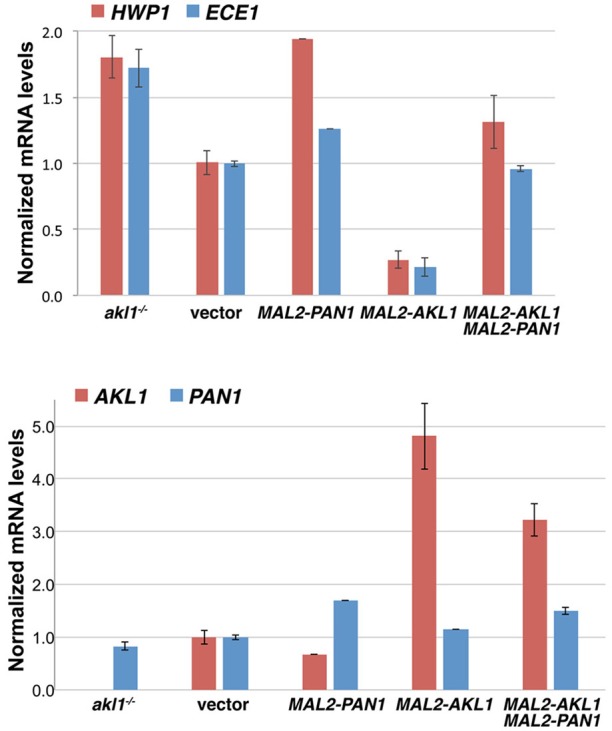
Effect of Akl1 and Pan1 on HSG expression. The strains used were the *akl1*^−/−^ deletion together with a vector plasmid (KC824), or wild-type with a vector plasmid (KC868), with *PAN1* under the *MAL2* promoter (KC869), with *AKL1* under the *MAL2* promoter (KC817), or with both *AKL1* and *PAN1* under the *MAL2* promoter (KC863). Overnight cultures were diluted in YEP-2% maltose and grown for 5 h at 37°C. Two independent cultures were assayed for each strain. The bar graph depicts the average of the two cultures. The Northern blots are shown in Supplementary Figure 8.

We tested the effect of *PAN1* overexpression and of *AKL1* overexpression and deletion on hyphal-specific gene (HSG) expression, using the representative HSGs *HWP1* and *ECE1*. As shown in Figure 8, *HWP1* and *ECE1*'s expression was well correlated with the morphology of the cells (Figures 1, 7): cells lacking *AKL1* or cells overexpressing *PAN1*, which showed a stronger hyphal morphogenesis, also exhibited higher HSG expression, whereas cells overexpressing *AKL1*, which showed a reduction in hyphal morphogenesis, exhibited lower HSG expression. Co-overexpression of *PAN1* and *AKL1* resulted in HSG expression similar to that of the control cells, consistent with the restored hyphal morphology observed in these cells (Figure 7). We also measured the *PAN1* and *AKL1* levels in the same cells, to confirm that their expression levels are increased as expected when expressed under the *MAL2* promoter. We found a 5-fold higher level of *AKL1* mRNA in cells containing an additional *AKL1* copy expressed under the *MAL2* promoter, whereas *PAN1* exhibited only 50% higher mRNA levels in the added presence of *MAL2-PAN1* in the cells.

## Discussion

In order to identify new post-translational repression mechanisms of fungal morphogenesis, we interrogated a comprehensive collection of kinases and phosphatases placed under the regulation of the TETon promoter. While this collection likely contains the vast majority of the kinase and phosphatase gene complement of *C. albicans*, it should be stressed that ectopic expression of a gene does not necessarily cause activation. Post-transcriptional control mechanisms, as well as post-translational mechanisms such as phosphorylation of MAP kinases, are often essential for full activation of a gene product. Thus it is likely that our screen only interrogated a fraction of the genes represented in the collection. Nonetheless, some general observations can be made. Most striking, a larger proportion of phosphatases was identified among the genes that inhibited hyphal morphogenesis. While this is consistent with the preponderant role that kinase-based signal transduction pathways play in the induction of hyphal morphogenesis, this result also highlights the potentially important but little-studied role that phosphatases may play in restricting hyphal induction under physiological conditions. Although a few examples are known of specific phosphatases that affect *C. albicans* hyphal morphogenesis, such as Sit4 (Lee et al., 2004) or the Protein Phosphatase 2A homolog Ppg1 (Albataineh et al., 2014), the relatively lower substrate specificity of phosphatases compared to kinases has traditionally made the roles of specific phosphatases harder to assess. It is possible that this lower specificity of phosphatases results in an overlap of target distribution of the phosphatases, causing loss-of-function deletion mutations to have fewer phenotypic effects. The screen presented here overcomes this problem by being based on ectopic gene overexpression and thus on gain of function, or more generally “dominant” effects. The results presented here are a useful reminder of the potential role of phosphatases in the regulation of fungal morphogenesis.

Among the kinases, several had previously been characterized as being involved in hyphal morphogenesis. We chose to focus on Akl1 because it was uncharacterized, exhibited one of the strongest effects on hyphal formation but did not affect yeast proliferation, and because the deletion of *AKL1* had the opposite effect, i.e., it enhanced hyphal morphogenesis. *Candida albicans AKL1* was found, like its *S. cerevisiae* counterpart, to regulate endocytosis. Another gene identified in our screen, *CLN3*, encodes an essential G1-specific cyclin of the main cell cycle kinase Cdc28. Interestingly, Cdc28-Cln3 had been previously linked to regulation of endocytosis and of hyphal development (Zeng et al., 2012), although it was also found to affect activity of the hyphal transcription factor Ume6 (Mendelsohn et al., 2017). A third gene identified in the screen, *VPS15*, encodes a regulator of endosome-to-Golgi retrograde transport (Liu et al., 2014), and could thus indirectly affect endocytosis as well.

The initial screen did not distinguish between suppression of germ tube induction vs. suppression of hyphal elongation. The kinetics were investigated in detail for the *AKL1*-overexpressing cells and the *akl1*^−/−^ cells, and found to specifically involve hyphal elongation rather than initiation. A role for endocytosis in *C. albicans* hyphal growth had previously been suggested by the observations that several mutants in actin regulatory complexes affected both fluid phase endocytosis and hyphal morphogenesis, including notably Pan1 (Martin et al., 2007), as well as myosin type I Myo5 (Oberholzer et al., 2002), the Wiscott-Aldrich Syndrome Protein homolog Wal1 (Walther and Wendland, 2004), the verprolin Vrp1 (Borth et al., 2010), the BAR domain proteins Rvs161 and Rvs167 (Douglas et al., 2009), and the clathrin adaptor protein Sla2 (Gale et al., 2009). *ARP2/3* deletions, which disrupt clathrin-mediated endocytosis while not affecting an alternative endocytic pathway, are also unable to form hyphae (Epp et al., 2010, 2013). The simplest explanation for a requirement for endocytosis in hyphal morphogenesis in general, is that the extensive exocytosis required for the rapid cell wall and cell membrane deposition at the tip of the extending hypha, needs to be counterbalanced by endocytosis in order to recycle the membranes and the proteins used in vesicle exocytosis (reviewed in Shaw et al., 2011). A higher requirement for endocytosis in hyphal growth, compared to yeast growth, would explain why the extent of suppression of endocytosis achieved by *AKL1* overexpression is sufficient to significantly depress hyphal elongation, but not to affect *C. albicans* yeast growth and proliferation. In further support of a higher requirement for endocytosis in hyphal growth, we found that drugs that suppress endocytosis also suppress hyphal elongation at concentrations at which yeast proliferation remains unimpeded (Bar-Yosef et al., 2017).

While our results underscore the previous reports indicating that inhibition of endocytosis suppresses hyphal growth, we find in addition that the enhancement of fluid-phase endocytosis measured in the *akl1*^−/−^ mutant and in cells overexpressing *PAN1*, are accompanied in both cases by accelerated hyphal elongation. This result was unexpected, and suggests that under standard hyphae induction conditions, endocytosis is limiting for optimal hyphal extension. We note however that, for the *akl1*^−/−^ mutant tested in YPD medium at 37°C, the large initial increase in germ tube extension vs. wild-type cells (60% longer hyphae in *akl1*^−/−^ after 1 h) is followed, during the second hour, by a smaller increase in hyphal elongation rates (24% faster elongation) in the absence of serum, or no difference at all in the presence of serum (Figure 3B). This observation suggests that under these conditions, the main contribution of the higher endocytosis rates of the *akl1*^−/−^ mutant may lie in “priming” the cells for hyphal elongation. At later time points, endocytosis might be physiologically accelerated in the wild-type, nullifying the initial advantage of the *akl1*^−/−^ strain, or alternatively, the larger hyphal cell bulk might make the relative endocytosis rate less rate-limiting for growth at the hyphal tip.

The faster germ tube extension in the *akl1*^−/−^ cells and in the *PAN1*-overexpressing cells is accompanied by increased expression of hyphal-specific genes, whereas the decreased germ tube extension in the *AKL1*-overexpressing cells is accompanied by decreased HSG expression. Our model however suggests that Akl1 affects morphogenesis via its effect on endocytosis, rather than on HSG expression. This is further supported by the observation that ectopic induction of hyphal morphogenesis by *UME6* expression, an inducer of HSGs, is blocked by *AKL1* overexpression, suggesting that Akl1 functions downstream of the hyphal induction pathway. However, if Akl1 functions downstream of the transcriptional regulation of hyphal morphogenesis, why then is HSG expression affected by Akl1? One possibility is that in addition to its endocytic pathway substrates such as Pan1, Akl1 also phosphorylates substrates in the hyphal induction pathway. However, this does not explain the effect of overexpression of Pan1 itself on both hyphal elongation and HSG expression. Therefore, it is more likely that it is the enhancement of endocytosis *per se*, and the ensuing enhancement of hyphal extension, that causes hyperinduction of HSG expression, and conversely, reduction of hyphal elongation via reduction in endocytosis causes decreased HSG expression. This would imply that beyond the transcriptional control of hyphal morphogenesis, the hyphal morphogenetic apparatus can regulate the hyphal gene expression program via a feedback mechanism. Such a feedback mechanism has been proposed previously between the GTPase exchange factor Cdc24 involved in hyphal growth and the hyphal transcription apparatus (Bassilana et al., 2005). The recent observation that interference with cellular physiology such as e.g., cell cycle progression, long known to induce filamentous growth, can also induce the HSG expression program even in the absence of external stimulation (Woolford et al., 2016), further supports the existence of such a feedback system. While the nature of this mechanism in *C. albicans* is still unknown, direct effects of cytoskeleton structure on transcription factor activity have been described in other systems (Posern et al., 2002; Miralles et al., 2003).

## Author contributions

Conceived and designed the experiments, HB-Y, TG, BR-Z, CS, JM, RA, and DK; Performed the experiments, HB-Y, TG, ZW, MP, RN, and DK; Analyzed the data, HB-Y, TG, ZW, and DK; Contributed reagents, materials, analysis tools, TG, BR-Z and CS; DK Wrote the paper, with input from all the authors, who read and approved the final draft of the manuscript.

### Conflict of interest statement

The authors declare that the research was conducted in the absence of any commercial or financial relationships that could be construed as a potential conflict of interest.

## References

[B1] Albataineh M. T. Lazzell A. Lopez-Ribot J. L. Kadosh D. (2014). Ppg1, a PP2A-type protein phosphatase, controls filament extension and virulence in *Candida albicans*. Eukaryotic Cell 13, 1538–1547. 10.1128/EC.00199-14 25326520 PMC4248689

[B2] Arana D. M. Nombela C. Alonso-Monge R. Pla J. (2005). The Pbs2 MAP kinase kinase is essential for the oxidative-stress response in the fungal pathogen *Candida albicans*. Microbiology 151, 1033–1049. 10.1099/mic.0.27723-0 15817773

[B3] Atir-Lande A. Gildor T. Kornitzer D. (2005). Role for the SCF(CDC4) ubiquitin ligase in *Candida albicans* morphogenesis. Mol. Biol. Cell 16, 2772–2785. 10.1091/mbc.E05-01-0079 15814839 PMC1142423

[B4] Bachewich C. Whiteway M. (2005). Cyclin Cln3p links G1 progression to hyphal and pseudohyphal development in *Candida albicans*. Eukaryotic Cell 4, 95–102. 10.1128/EC.4.1.95-102.2005 15643065 PMC544164

[B5] Banerjee M. Thompson D. S. Lazzell A. Carlisle P. L. Pierce C. Monteagudo C.. (2008). UME6, a novel filament-specific regulator of *Candida albicans* hyphal extension and virulence. Mol. Biol. Cell 19, 1354–1365. 10.1091/mbc.E07-11-1110 18216277 PMC2291399

[B6] Bar-Yosef H. Vivanco Gonzalez N. Ben-Aroya S. Kron S. J. Kornitzer D. (2017). Chemical inhibitors of *Candida albicans* hyphal morphogenesis target endocytosis. Sci. Rep. 7:5692. 10.1038/s41598-017-05741-y 28720834 PMC5515890

[B7] Basrai M. A. Naider F. Becker J. M. (1990). Internalization of lucifer yellow in *Candida albicans* by fluid phase endocytosis. J. Gen. Microbiol. 136, 1059–1065. 10.1099/00221287-136-6-1059 2200841

[B8] Bassilana M. Hopkins J. Arkowitz R. A. (2005). Regulation of the Cdc42/Cdc24 GTPase module during *Candida albicans* hyphal growth. Eukaryotic Cell 4, 588–603. 10.1128/EC.4.3.588-603.2005 15755921 PMC1087799

[B9] Biswas K. Morschhäuser J. (2005). The Mep2p ammonium permease controls nitrogen starvation-induced filamentous growth in *Candida albicans*. Mol. Microbiol. 56, 649–669. 10.1111/j.1365-2958.2005.04576.x 15819622

[B10] Borth N. Walther A. Reijnst P. Jorde S. Schaub Y. Wendland J. (2010). *Candida albicans* Vrp1 is required for polarized morphogenesis and interacts with Wal1 and Myo5. Microbiology 156, 2962–2969. 10.1099/mic.0.041707-0 20656786

[B11] Bradford M. K. Whitworth K. Wendland B. (2015). Pan1 regulates transitions between stages of clathrin-mediated endocytosis. Mol. Biol. Cell 26, 1371–1385. 10.1091/mbc.E14-11-1510 25631817 PMC4454182

[B12] Braun B. R. Kadosh D. Johnson A. D. (2001). NRG1, a repressor of filamentous growth in *C.albicans*, is down-regulated during filament induction. EMBO J. 20, 4753–4761. 10.1093/emboj/20.17.4753 11532939 PMC125265

[B13] Brown G. D. Denning D. W. Gow N. A. Levitz S. M. Netea M. G. White T. C. (2012). Hidden killers: human fungal infections. Sci. Transl. Med. 4:165rv113. 10.1126/scitranslmed.3004404 23253612

[B14] Carlisle P. L. Banerjee M. Lazzell A. Monteagudo C. LLópez-Ribot J. L. Kadosh D. (2009). Expression levels of a filament-specific transcriptional regulator are sufficient to determine *Candida albicans* morphology and virulence. Proc. Natl. Acad. Sci. U.S.A. 106, 599–604. 10.1073/pnas.0804061106 19116272 PMC2626749

[B15] Chapa Y Lazo B. Bates S. Sudbery P. (2005). The G1 cyclin Cln3 regulates morphogenesis in *Candida albicans*. Eukaryotic Cell 4, 90–94. 10.1128/EC.4.1.90-94.2005 15643064 PMC544163

[B16] Collart M. A. Oliviero S. (2001). Preparation of yeast RNA. Curr. Protoc. Mol. Biol. Chapter 13, Unit13 12. 10.1002/0471142727.mb1312s23 18265096

[B17] Corvey C. Koetter P. Beckhaus T. Hack J. Hofmann S. Hampel M.. (2005). Carbon Source-dependent assembly of the Snf1p kinase complex in *Candida albicans*. J. Biol. Chem. 280, 25323–25330. 10.1074/jbc.M503719200 15890650

[B18] Cross F. R. (1988). DAF1, a mutant gene affecting size control, pheromone arrest, and cell cycle kinetics of *Saccharomyces cerevisiae*. Mol. Cell. Biol. 8, 4675–4684. 10.1128/MCB.8.11.4675 3062366 PMC365557

[B19] Di Tommaso P. Moretti S. Xenarios I. Orobitg M. Montanyola A. Chang J. M.. (2011). T-Coffee: a web server for the multiple sequence alignment of protein and RNA sequences using structural information and homology extension. Nucleic Acids Res. 39, W13–W17. 10.1093/nar/gkr245 21558174 PMC3125728

[B20] Douglas L. M. Martin S. W. Konopka J. B. (2009). BAR domain proteins Rvs161 and Rvs167 contribute to *Candida albicans* endocytosis, morphogenesis, and virulence. Infect. Immun. 77, 4150–4160. 10.1128/IAI.00683-09 19596778 PMC2738028

[B21] Dulic V. Egerton M. Elguindi I. Raths S. Singer B. Riezman H. (1991). Yeast endocytosis assays. Meth. Enzymol. 194, 697–710. 10.1016/0076-6879(91)94051-D 2005817

[B22] Elson S. L. Noble S. M. Solis N. V. Filler S. G. Johnson A. D. (2009). An RNA transport system in *Candida albicans* regulates hyphal morphology and invasive growth. PLoS Genet. 5:e1000664. 10.1371/journal.pgen.1000664 19779551 PMC2739428

[B23] Epp E. Nazarova E. Regan H. Douglas L. M. Konopka J. B. Vogel J.. (2013). Clathrin- and Arp2/3-independent endocytosis in the fungal pathogen *Candida albicans*. MBio 4:e00476-13. 10.1128/mBio.00476-13 23982070 PMC3760247

[B24] Epp E. Walther A. Lepine G. Leon Z. Mullick A. Raymond M. (2010). Forward genetics in *Candida albicans* that reveals the Arp2/3 complex is required for hyphal formation, but not endocytosis. Mol. Microbiol. 75, 1182–1198. 10.1111/j.1365-2958.2009.07038.x 20141603 PMC4092012

[B25] Feng Q. Summers E. Guo B. Fink G. (1999). Ras signaling is required for serum-induced hyphal differentiation in *Candida albicans*. J. Bacteriol. 181, 6339–6346. 10515923 10.1128/jb.181.20.6339-6346.1999 PMC103768

[B26] Fonzi W. A. Irwin M. Y. (1993). Isogenic strain construction and gene mapping in *Candida albicans*. Genetics 134, 717–728. 8349105 10.1093/genetics/134.3.717 PMC1205510

[B27] Gale C. A. Leonard M. D. Finley K. R. Christensen L. McClellan M. Abbey D.. (2009). SLA2 mutations cause SWE1-mediated cell cycle phenotypes in *Candida albicans* and *Saccharomyces cerevisiae*. Microbiology 155, 3847–3859. 10.1099/mic.0.033233-0 19778960 PMC2846636

[B28] Gildor T. Shemer R. Atir-Lande A. Kornitzer D. (2005). Coevolution of cyclin Pcl5 and its substrate Gcn4. Eukaryotic Cell 4, 310–318. 10.1128/EC.4.2.310-318.2005 15701793 PMC549342

[B29] Gola S. Martin R. Walther A. Dünkler A. Wendland J. (2003). New modules for PCR-based gene targeting in *Candida albicans*: rapid and efficient gene targeting using 100 bp of flanking homology region. Yeast 20, 1339–1347. 10.1002/yea.1044 14663826

[B30] Hanaoka N. Takano Y. Shibuya K. Fugo H. Uehara Y. Niimi M. (2008). Identification of the putative protein phosphatase gene PTC1 as a virulence-related gene using a silkworm model of *Candida albicans* infection. Eukaryotic Cell 7, 1640–1648. 10.1128/EC.00129-08 18708562 PMC2568064

[B31] Hu K. Li W. Wang H. Chen K. Wang Y. Sang J. (2012). Shp1, a regulator of protein phosphatase 1 Glc7, has important roles in cell morphogenesis, cell cycle progression and DNA damage response in *Candida albicans*. Fungal Genet. Biol. 49, 433–442. 10.1016/j.fgb.2012.04.004 22542681

[B32] Hunter T. Plowman G. (1997). The protein kinases of budding yeast: six score and more. Trends Biochem. Sci. 22, 18–22. 10.1016/S0968-0004(96)10068-2 9020587

[B33] Kaksonen M. Sun Y. Drubin D. G. (2003). A pathway for association of receptors, adaptors, and actin during endocytic internalization. Cell 115, 475–487. 10.1016/S0092-8674(03)00883-3 14622601

[B34] Kaksonen M. Toret C. P. Drubin D. G. (2005). A modular design for the clathrin- and actin-mediated endocytosis machinery. Cell 123, 305–320. 10.1016/j.cell.2005.09.024 16239147

[B35] Kempf C. Bathe F. Fischer R. (2013). Evidence that two Pcl-like cyclins control Cdk9 activity during cell differentiation in *Aspergillus nidulans* asexual development. Eukaryotic Cell 12, 23–36. 10.1128/EC.00181-12 23104571 PMC3535853

[B36] Kornitzer D. (2002). Monitoring protein degradation. Meth. Enzymol. 351, 639–647. 10.1016/S0076-6879(02)51874-7 12073374

[B37] Leberer E. Harcus D. Broadbent I. D. Clark K. L. Dignard D. Ziegelbauer K.. (1996). Signal transduction through homologs of the Ste20p and Ste7p protein kinases can trigger hyphal formation in the pathogenic fungus *Candida albicans*. Proc. Natl. Acad. Sci. U.S.A. 93, 13217–13222. 10.1073/pnas.93.23.13217 8917571 PMC24073

[B38] Lee C. M. Nantel A. Jiang L. Whiteway M. Shen S. H. (2004). The serine/threonine protein phosphatase SIT4 modulates yeast-to-hypha morphogenesis and virulence in *Candida albicans*. Mol. Microbiol. 51, 691–709. 10.1111/j.1365-2958.2003.03879.x 14731272

[B39] Liu Y. Solis N. V. Heilmann C. J. Phan Q. T. Mitchell A. P. Klis F. M.. (2014). Role of retrograde trafficking in stress response, host cell interactions, and virulence of *Candida albicans*. Eukaryotic Cell 13, 279–287. 10.1128/EC.00295-13 24363364 PMC3910971

[B40] Lu Y. Su C. Unoje O. Liu H. (2014). Quorum sensing controls hyphal initiation in *Candida albicans* through Ubr1-mediated protein degradation. Proc. Natl. Acad. Sci. U.S.A. 111, 1975–1980. 10.1073/pnas.1318690111 24449897 PMC3918812

[B41] Lu Y. Su C. Wang A. Liu H. (2011). Hyphal development in *Candida albicans* requires two temporally linked changes in promoter chromatin for initiation and maintenance. PLoS Biol. 9:e1001105. 10.1371/journal.pbio.1001105 21811397 PMC3139633

[B42] Maidan M. M. De Rop L. Serneels J. Exler S. Rupp S. Tournu H.. (2005). The G protein-coupled receptor Gpr1 and the Galpha protein Gpa2 act through the cAMP-protein kinase A pathway to induce morphogenesis in *Candida albicans*. Mol. Biol. Cell 16, 1971–1986. 10.1091/mbc.E04-09-0780 15673611 PMC1073676

[B43] Manning G. Whyte D. B. Martinez R. Hunter T. Sudarsanam S. (2002). The protein kinase complement of the human genome. Science 298, 1912–1934. 10.1126/science.1075762 12471243

[B44] Martin R. Hellwig D. Schaub Y. Bauer J. Walther A. Wendland J. (2007). Functional analysis of *Candida albicans* genes whose *Saccharomyces cerevisiae* homologues are involved in endocytosis. Yeast 24, 511–522. 10.1002/yea.1489 17431925

[B45] Mendelsohn S. Pinsky M. Weissman Z. Kornitzer D. (2017). Regulation of the *Candida albicans* hypha-inducing transcription factor Ume6 by the CDK1 cyclins Cln3 and Hgc1. mSphere 2:e00248-16. 10.1128/mSphere.00248-16 28289726 PMC5343172

[B46] Miralles F. Posern G. Zaromytidou A. I. Treisman R. (2003). Actin dynamics control SRF activity by regulation of its coactivator MAL. Cell 113, 329–342. 10.1016/S0092-8674(03)00278-2 12732141

[B47] Murad A. M. Leng P. Straffon M. Wishart J. Macaskill S. MacCallum D.. (2001). NRG1 represses yeast-hypha morphogenesis and hypha-specific gene expression in *Candida albicans*. EMBO J. 20, 4742–4752. 10.1093/emboj/20.17.4742 11532938 PMC125592

[B48] Nash R. Tokiwa G. Anand S. Erickson K. Futcher A. B. (1988). The WHI1+ gene of *Saccharomyces cerevisiae* tethers cell division to cell size and is a cyclin homolog. EMBO J. 7, 4335–4346. 2907481 10.1002/j.1460-2075.1988.tb03332.x PMC455150

[B49] Noble S. M. Johnson A. D. (2005). Strains and strategies for large-scale gene deletion studies of the diploid human fungal pathogen *Candida albicans*. Eukaryotic Cell 4, 298–309. 10.1128/EC.4.2.298-309.2005 15701792 PMC549318

[B50] Oberholzer U. Marcil A. Leberer E. Thomas D. Y. Whiteway M. (2002). Myosin I is required for hypha formation in *Candida albicans*. Eukaryotic Cell 1, 213–228. 10.1128/EC.1.2.213-228.2002 12455956 PMC118025

[B51] Ofir A. Hofmann K. Weindling E. Gildor T. Barker K. S. Rogers P. D.. (2012). Role of a *Candida albicans* Nrm1/Whi5 homologue in cell cycle gene expression and DNA replication stress response. Mol. Microbiol. 84, 778–794. 10.1111/j.1365-2958.2012.08056.x 22463761 PMC3345080

[B52] Posern G. Sotiropoulos A. Treisman R. (2002). Mutant actins demonstrate a role for unpolymerized actin in control of transcription by serum response factor. Mol. Biol. Cell 13, 4167–4178. 10.1091/mbc.02-05-0068 12475943 PMC138624

[B53] Ramírez-Zavala B. Mottola A. Haubenreißer J. Schneider S. Allert S. Brunke S.. (2017). The Snf1-activating kinase Sak1 is a key regulator of metabolic adaptation and *in vivo* fitness of *Candida albicans*. Mol. Microbiol. 104, 989–1007. 10.1111/mmi.13674 28337802

[B54] Ramírez-Zavala B. Weyler M. Gildor T. Schmauch C. Kornitzer D. Arkowitz R.. (2013). Activation of the Cph1-dependent MAP kinase signaling pathway induces white-opaque switching in *Candida albicans*. PLoS Pathog. 9:e1003696. 10.1371/journal.ppat.1003696 24130492 PMC3795047

[B55] Seger R. Seger D. Reszka A. A. Munar E. S. Eldar-Finkelman H. Dobrowolska G.. (1994). Overexpression of mitogen-activated protein kinase kinase (MAPKK) and its mutants in NIH 3T3 cells. Evidence that MAPKK involvement in cellular proliferation is regulated by phosphorylation of serine residues in its kinase subdomains VII and VIII. J. Biol. Chem. 269, 25699–25709. 7929275

[B56] Shaw B. D. Chung D. W. Wang C. L. Quintanilla L. A. Upadhyay S. (2011). A role for endocytic recycling in hyphal growth. Fungal Biol. 115, 541–546. 10.1016/j.funbio.2011.02.010 21640317

[B57] Smythe E. Ayscough K. R. (2003). The Ark1/Prk1 family of protein kinases. Regulators of endocytosis and the actin skeleton. EMBO Rep. 4, 246–251. 10.1038/sj.embor.embor776 12634840 PMC1315904

[B58] Stoldt V. R. Sonneborn A. Leuker C. E. Ernst J. F. (1997). Efg1p, an essential regulator of morphogenesis of the human pathogen *Candida albicans*, is a member of a conserved class of bHLH proteins regulating morphogenetic processes in fungi. EMBO J. 16, 1982–1991. 10.1093/emboj/16.8.1982 9155024 PMC1169801

[B59] Sudbery P. Gow N. Berman J. (2004). The distinct morphogenic states of *Candida albicans*. Trends Microbiol. 12, 317–324. 10.1016/j.tim.2004.05.008 15223059

[B60] Sun L. L. Li W. J. Wang H. T. Chen J. Deng P. Wang Y.. (2011). Protein phosphatase Pph3 and its regulatory subunit Psy2 regulate Rad53 dephosphorylation and cell morphogenesis during recovery from DNA damage in *Candida albicans*. Eukaryotic Cell 10, 1565–1573. 10.1128/EC.05042-11 21890819 PMC3209060

[B61] Sun W. Kesavan K. Schaefer B. C. Garrington T. P. Ware M. Johnson N. L.. (2001). MEKK2 associates with the adapter protein Lad/RIBP and regulates the MEK5-BMK1/ERK5 pathway. J. Biol. Chem. 276, 5093–5100. 10.1074/jbc.M003719200 11073940

[B62] Sun Y. Leong N. T. Wong T. Drubin D. G. (2015). A Pan1/End3/Sla1 complex links Arp2/3-mediated actin assembly to sites of clathrin-mediated endocytosis. Mol. Biol. Cell 26, 3841–3856. 10.1091/mbc.E15-04-0252 26337384 PMC4626068

[B63] Takahashi T. Furuchi T. Naganuma A. (2006). Endocytic Ark/Prk kinases play a critical role in adriamycin resistance in both yeast and mammalian cells. Cancer Res. 66, 11932–11937. 10.1158/0008-5472.CAN-06-3220 17178891

[B64] Uhl M. A. Biery M. Craig N. Johnson A. D. (2003). Haploinsufficiency-based large-scale forward genetic analysis of filamentous growth in the diploid human fungal pathogen C.albicans. EMBO J. 22, 2668–2678. 10.1093/emboj/cdg256 12773383 PMC156753

[B65] Umeyama T. Kaneko A. Nagai Y. Hanaoka N. Tanabe K. Takano Y.. (2005). Candida albicans protein kinase CaHsl1p regulates cell elongation and virulence. Mol. Microbiol. 55, 381–395. 10.1111/j.1365-2958.2004.04405.x 15659158

[B66] Walther A. Wendland J. (2004). Polarized hyphal growth in *Candida albicans* requires the Wiskott-Aldrich syndrome protein homolog Wal1p. Eukaryotic Cell 3, 471–482. 10.1128/EC.3.2.471-482.2004 15075276 PMC387638

[B67] Watters M. K. Boersma M. Johnson M. Reyes C. Westrick E. Lindamood E. (2011). A screen for Neurospora knockout mutants displaying growth rate dependent branch density. Fungal Biol. 115, 296–301. 10.1016/j.funbio.2010.12.015 21354536

[B68] Wendland B. Emr S. D. (1998). Pan1p, yeast eps15, functions as a multivalent adaptor that coordinates protein-protein interactions essential for endocytosis. J. Cell Biol. 141, 71–84. 10.1083/jcb.141.1.71 9531549 PMC2132731

[B69] Wightman R. Bates S. Amornrrattanapan P. Sudbery P. (2004). in *Candida albicans*, the Nim1 kinases Gin4 and Hsl1 negatively regulate pseudohypha formation and Gin4 also controls septin organization. J. Cell Biol. 164, 581–591. 10.1083/jcb.200307176 14769857 PMC2171991

[B70] Woolford C. A. Lagree K. Xu W. Aleynikov T. Adhikari H. Sanchez H.. (2016). Bypass of *Candida albicans* filamentation/biofilm regulators through diminished expression of protein kinase Cak1. PLoS Genet. 12:e1006487. 10.1371/journal.pgen.1006487 27935965 PMC5147786

[B71] Zeidler U. Lettner T. Lassnig C. Müller M. Lajko R. Hintner H.. (2009). UME6 is a crucial downstream target of other transcriptional regulators of true hyphal development in *Candida albicans*. FEMS Yeast Res. 9, 126–142. 10.1111/j.1567-1364.2008.00459.x 19054126

[B72] Zeng G. Wang Y. M. Wang Y. (2012). Cdc28-Cln3 phosphorylation of Sla1 regulates actin patch dynamics in different modes of fungal growth. Mol. Biol. Cell 23, 3485–3497. 10.1091/mbc.E12-03-0231 22787279 PMC3431942

[B73] Zhang C. Konopka J. B. (2010). A photostable green fluorescent protein variant for analysis of protein localization in *Candida albicans*. Eukaryotic Cell 9, 224–226. 10.1128/EC.00327-09 19915075 PMC2805285

