# Supplementary material for: A Global Analysis of Kinase Function in Candida albicans Hyphal Morphogenesis Reveals a Role for the Endocytosis Regulator Akl1
Source: Front Cell Infect Microbiol. 2018 Feb 8;8:17. doi: 10.3389/fcimb.2018.00017 (PMC5809406; doi:10.3389/fcimb.2018.00017)
Supplement: Supplementary file 1 [file Image1.pdf]

# *Supplementary material*
# A Global Analysis of Kinase Function in *Candida albicans* Hyphal Morphogenesis Reveals a Role for the Endocytosis Regulator Akl1

Hagit Bar-Yosef[1,4], Tsvia Gildor[1,4], Bernardo Ramírez-Zavala[2], Christian Schmauch[3], Ziva Weissman[1], Mariel Pinsky[1], Rawi Naddaf[1], Joachim Morschhäuser[2], Robert A. Arkowitz[3] and Daniel Kornitzer[1]*
* Corresponding author. e-mail: danielk@technion.ac.il

## Supplementary Figures

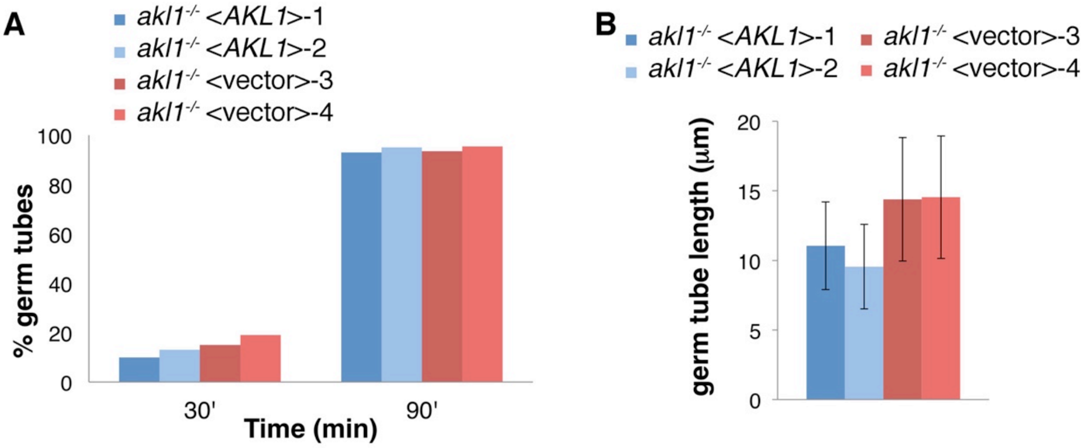


**Supplementary Figure 1. The rate of hyphal elongation is increased in the *akl1*$^{-/-}$ mutant.** A. Germ tube induction in strains of the *akl1*$^{-/-}$ mutant transformed with the vector plasmid (KC824) vs. the *AKL1* reintegrant plasmid (KC825), two independent transformants each (KC824: clones 3 and 4, KC825: clones 1 and 2). Cells were grown overnight in YPD at 30°C, then diluted into fresh YPD and shifted to 37°C. Samples were retrieved and fixed at the indicated times and the proportion of germ tubes was determined by microscopic observation. 200 cells were counted in each culture. B. Length of germ tubes was measured after 90 min in the same cultures as for panel A. 100 cells were measured for each condition. The error bars indicate the standard deviations. The differences between each of the mutant vs. each of the reintegrant strains were all highly significant by Student's two-tailed *t*-test ($p = 2x10^{-9}$ and lower).

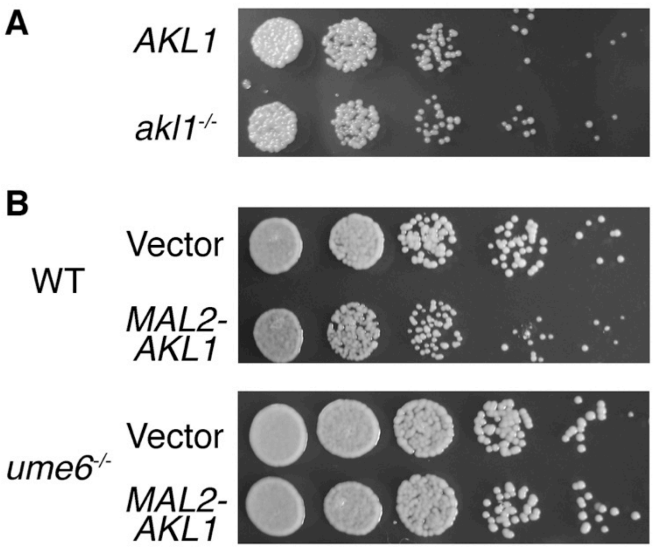


**Supplementary Figure 2. *AKL1* deletion or overexpression does not affect the growth rate.** A. Drop dilution of wild-type (KC274) and *akl1*$^{-/-}$ (KC554) on YPD. Plates were incubated 1 day at 37°C. B. Top panel: Drop dilution of wild-type cells carrying the vector plasmid (KC840) or *MAL2-AKL1* (KC841) on YEP + 2% maltose. Plates were incubated 1 day at 37°C. Since the slight change in appearance of the colonies could be due hyphal cell morphology rather than differences in cell growth, we also tested the effect of *MAL2-AKL1* on growth of the non-filamentous *ume6*$^{-/-}$ mutant (KC445; bottom panel).

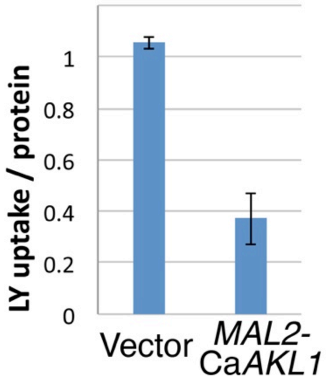


**Supplementary Figure 3. CaAkl1 represses fluid-phase endocytosis in the Ca*ume6*$^{-/-}$ mutant.** LY uptake in *ume6*$^{-/-}$ cells overexpressing *AKL1* under the *MAL2* promoter (KC851) *vs.* a vector plasmid (KC850). Overnight cultures were shifted from YEP + 2% raffinose to YEP + 2% maltose and incubated at 37°C for 2.5 h before carrying out the assays. The error bars indicate the standard deviation between three independent cultures.

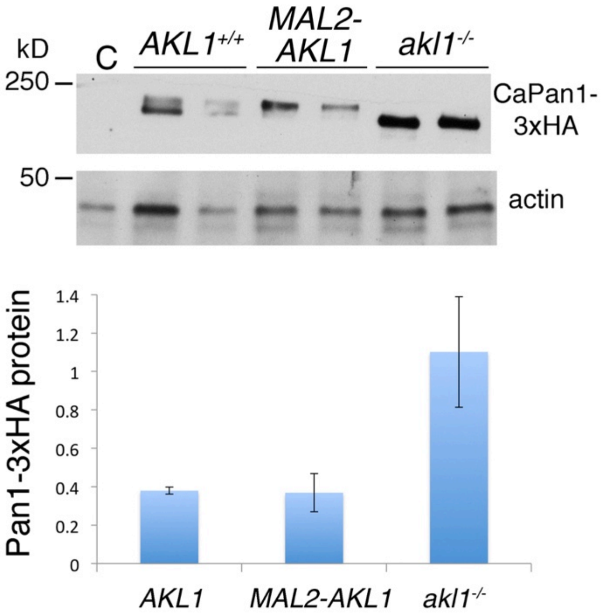


**Supplementary Figure 4. Quantitation of CaPan1 in cells lacking or overexpressing *AKL1*.** The same extracts used in Fig. 5A were migrated on a 4-20% gradient SDS-PAGE gel. The membrane was probed with the anti-HA antibody and with an anti-actin antibody. The lower panel represents the HA signal divided for each lane by its actin signal (average of the two extracts).

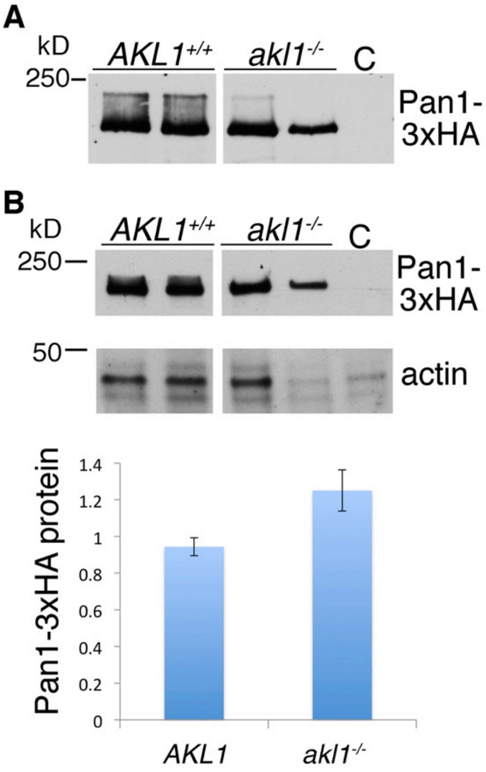


**Supplementary Figure 5. Induction of post-translational modification of Pan1 by Akl1 in glucose.** A. Western blot analysis of Pan1-3xHA expressed under its endogenous promoter in wild-type (KC983) and *akl1*$^{-/-}$ (KC984) cells (two cultures each). Cells were grown in YPD at 37°C for 4 h. The extracts were migrated on a 6%

SDS-PAGE gel, and the membrane was probed with the anti-HA antibody. B. The same extracts were migrated on a 4-20% SDS-PAGE gel, and the membrane was probed with the anti-HA antibody and with an anti-actin antibody. The lower panel represents the HA signal divided for each lane by its actin signal (average of the two extracts).

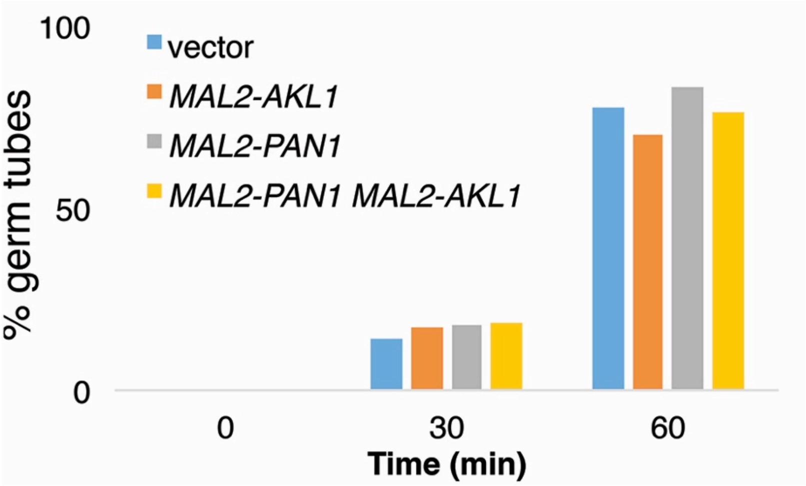


**Supplementary Figure 6. Effect of *MAL2*-AKL1 and *MAL2*-PAN1 on germ tube induction.** The cultures described in Fig. 7 were fixed at the indicated times and the proportion of germ tubes was determined by microscopic observation. 200 cells were counted in each culture.

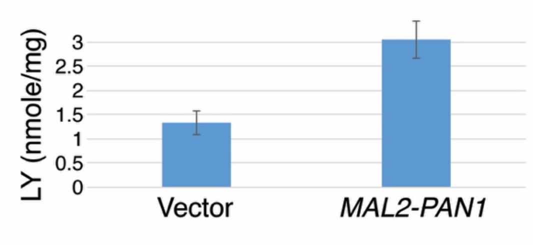


**Supplementary Figure 7. LY uptake induction by Pan1 in yeast cells at 37°C.** Overnight cultures of *ume6*$^{-/-}$ cells overexpressing *PAN1* under the *MAL2* promoter (KC988) *vs.* carrying a vector plasmid (KC987)were shifted from YEP + 2% raffinose to YEP + 2% maltose and incubated at 37°C for 2.5 h before carrying out the assays. The error bars indicate the standard deviation between three independent cultures.

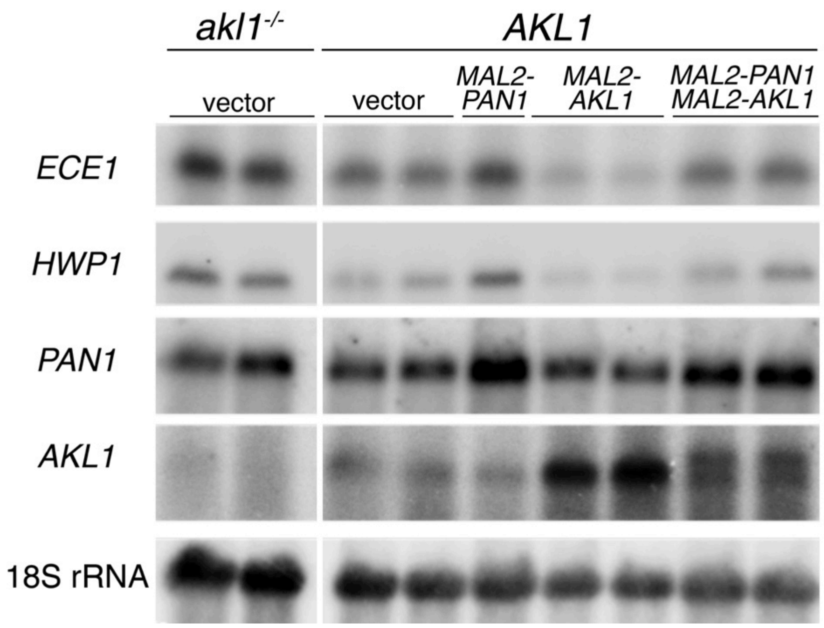


**Supplementary Figure 8**. **Northern blot hybridization analysis of the HSGs in the absence of *AKL1* or upon overexpression of *AKL1* and of *PAN1*.** Band intensity was quantitated by Phosphorimager. Quantitation is shown in Fig. 8.

## Supplementary Tables

**Supplementary Table** 1:

Strain WO1 carrying the indicated genes under TETon regulation were grown overnight in YPD + doxycyclin, then diluted into YPD + doxycyclin + 10% fetal calf serum, and visualized under the microscope after 5.5 hours incubation at 30°C. Under these conditions, the control strain exhibited 25%-30% hyphae. The morphology was scored as follows: 5% = -4, 10% = -3, 15% = -2, 20%=-1, 25-30% = 0, 40%=1, 50%=2, 60-70%=3, 80-100%=4. For each clone, scores of three cultures were averaged.
